# Insight Into the Role of PC_71_BM on Enhancing the Photovoltaic Performance of Ternary Organic Solar Cells

**DOI:** 10.3389/fchem.2018.00198

**Published:** 2018-06-05

**Authors:** Bei Wang, Yingying Fu, Chi Yan, Rui Zhang, Qingqing Yang, Yanchun Han, Zhiyuan Xie

**Affiliations:** ^1^State Key Laboratory of Polymer Physics and Chemistry, Changchun Institute of Applied Chemistry, Chinese Academy of Sciences, Changchun, China; ^2^University of Science and Technology of China, Hefei, China; ^3^University of Chinese Academy of Sciences, Beijing, China

**Keywords:** ternary organic solar cells, morphology, aggregation, charge transport, trap density

## Abstract

The development of non-fullerene acceptor molecules have remarkably boosted power conversion efficiency (PCE) of polymer solar cells (PSCs) due to the improved spectral coverage and reduced energy loss. An introduction of fullerene molecules into the non-fullerene acceptor-based blend may further improve the photovoltaic performance of the resultant ternary PSCs. However, the underlying mechanism is still debatable. Herein, the ternary PSCs based on PBDB-T:ITIC:PC_71_BM blend were fabricated and its PCE was increased to 10.2% compared to 9.2% for the binary PBDB-T:ITIC devices and 8.1% for the PBDB-T:PC_71_BM PSCs. Systematic investigation was carried out to disclose the effect of PC_71_BM on the blend morphology and charge transport behavior. It is found that the PC_71_BM tends to intermix with the PBDB-T donor compared to the ITIC counterpart. A small amount of PC_71_BM in the ternary blend is helpful for ITIC to aggregate and form efficient electron-transport pathways. Accordingly, the electron mobility is increased and the density of electron traps is decreased in the ternary blend in comparison with the PBDB-T:ITIC blend. Finally, the suppressed bimolecular recombination and enhanced charge collection lead to high PCE for the ternary solar cells.

## Introduction

Non-fullerene acceptors have drawn great research interests in the community of polymer solar cells (PSCs) in recent years. The power conversion efficiencies (PCE) of PSCs employing non-fullerene acceptors have increased rapidly as compared to the PSCs using fullerene derivatives as acceptors (Lin et al., 2015; Cao et al., 2017; Fan et al., 2017; Li et al., 2017; Xiao et al., 2017; Dai et al., 2018). Non-fullerene acceptors possess some advantages such as strong absorption in the visible region and tunable energy levels with regard to fullerene derivatives, and thus allows for suitable combination of donor/acceptor blend to improve the spectral coverage and reduce the energy loss (Holliday et al., 2016; Li et al., 2016, 2018; Qiu et al., 2017). In addition to the strong and complementary absorption and the matched energy levels of the donor/acceptor combination, the donor/acceptor morphology with a suitable phase separation is equally important for realizing a high PCE. More recently, addition of another type of donor or acceptor in the binary non-fullerene PSCs to fabricate so-called ternary PSCs have drawn great interests. This kind of ternary strategy is to some extent powerful to enhance the photovoltaic performance of the devices (Cheng et al., 2014; Gasparini et al., 2016). As it is argued, ternary PSCs possess some features such as more complementary absorption (Jiang et al., 2018) and more appropriate microstructure relative to the binary counterparts (Wang et al., 2017) and easier fabrication compared with tandem solar cells. With these superiorities, ternary PSCs have developed very quickly and become a research focus in the field (Yu et al., 2017; Zhang G. et al., 2017; Zhao et al., 2017; Wang et al., 2018). Nonetheless, the morphology of ternary blends is more complex resulting in the underlying mechanism debatable. It is proposed that the ternary morphology can be divided into four types according to the relative position of third component to the donor phases and acceptor phases, namely the third component embedded in one phase, located at the interfaces, formed alloy structure with either the donor or acceptor material and parallel-like bulk heterojunction structure with the donor or acceptor (Lu et al., 2015). In fact, the ternary morphology is too complicated to be clearly identified using the current technology. Typical electron acceptor fullerene derivatives, such as PC_71_BM and Bis-PC_71_BM have been used as additives in non-fullerene PSCs. For example, Bo et al. have reported high-performance ternary PSCs employing non-fullerene and fullerene acceptors simultaneously for the first time, in which they found that a small amount of fullerene is in favor of boosting the photovoltaic performance of the devices (Lu et al., 2016). Hou et al. also reported the high-efficiency ternary PSCs using Bis-PC_71_BM as the third component (Zhao et al., 2017). They proposed that Bis-PC_71_BM mainly exists in the upper surface of active layer and promotes electron transport in their ternary blend PSCs. For the ternary PSCs, the third component such as PC_71_BM may have strong effect on the resultant ternary morphology and hence its photovoltaic properties. Although some researches on the function of third component were carried out (Yu et al., 2017; Zhang J. et al., 2017; Wang et al., 2018), the underlying mechanism is still debatable.

Herein, a reported wide bandgap polymer poly[(2,6- (4,8-bis(5-(2-ethylhexyl)thiophen-2-yl)-benzo[1,2-b:4,5-b′] dithiophene))-alt-(5,5-(1′,3′-di-2-thienyl-5′,7′-bis(2-ethylhexyl) benzo[1′,2′-c:4′,5′-c′]dithiophene-4,8-dione))] (PBDB-T) is used as the donor, organic molecule (3,9-bis(2-methylene-(3- (1,1-dicyanomethylene)-indanone))- 5,5,11,11-tetrakis(4-hexylphenyl)-dithieno[2,3-d:2′,3′-d′]-s-indaceno[1,2-b:5,6-b′] dithiophene) (ITIC) is used as the acceptor and [6,6]-phenyl-C71-butyric acid methyl ester (PC_71_BM) is used as the third component to prepare the ternary PSCs. The optimized PSCs based on ternary PBDB-T:ITIC:PC_71_BM blend demonstrate a higher PCE of 10.2% than 9.2% of the binary PBDB-T:ITIC devices and 8.1% of the PBDB-T:PC_71_BM PSCs. Further studies are mainly focused on the effect of PC_71_BM on the blend morphology and charge transport behavior. It is found that the PC_71_BM tends to intermix with the PBDB-T donor compared to the ITIC counterpart. A small amount of PC_71_BM in the ternary blend is helpful for ITIC to aggregate and form efficient electron-transport pathways. Accordingly, the electron mobility is increased and the density of electron traps is decreased in the ternary blend in comparison with the PBDB-T:ITIC blend. Finally, the suppressed bimolecular recombination and enhanced charge collection lead to an enhanced PCE for the ternary solar cells.

## Experimental section

Both the polymer PBDB-T donor and small molecule ITIC acceptor were bought from Solarmer Ltd. The PBDB-T has a molecular weight Mn of 21.5 kDa and a PDI of 1.9. PC_71_BM was bought from American Dye Source. The interfacial material PDINO was provided by Dr. Zhiguo Zhang in Institute of Chemistry, Chinese Academy of Sciences. All materials were used as received without further purification.

Polymer solar cells were fabricated with a structure of ITO/PEDOT: PSS/active layer/PDINO/Al. The ITO substrates were subject to routine cleaning procedure of detergent, acetone and deionized water. After drying in an oven for 30 min, the ITO substrates were treated with UV-ozone for 25 min. The PEDOT:PSS layer was first deposited via spin-coating and dried at 140°C for 30 min in air. The subsequent active layer and buffer layer were spin-coated in a glove box. The active layers consisting of PBDB-T:PC_71_BM, PBDB-T:ITIC, or ternary PBDB-T:ITIC:PC_71_BM were spin-coated from their respective solutions in CB containing 0.5% DIO with a total concentration of 20 mg/mL. The spin-coating rate for the active layers was kept at 2,500 rpm for 1 min. The samples were annealed at 160°C for 10 min. A cathode buffer layer of PDINO was spin-coated on the active layer from its methanol solution with a concentration of 1 mg/mL at 3,000 rpm for 30 s. Finally, the Al cathode with a thickness of 100 nm was thermally deposited in a vacuum chamber. The electron—and hole-only devices were fabricated under the same procedure with a structure of ITO/PEIE/active layer/PDINO/Al and ITO/PEDOT:PSS/active layer/MoO_3_/Al, respectively.

The current density-voltage (J-V) curves of the PSCs were traced by a computer-controlled Keithley 2400 Source Meter under simulated solar light illumination (AM 1.5G, 100 mW/cm^2^). The EQE data were measured by solar cell spectral response measurement system (QE-R 3011, Enli Tech. Co.). The film absorption and fluorescence spectra were recorded on Agilent Cary 60UV-Vis spectrophotometer and Perkin-Elmer LS 55 spectrofluorometer, respectively. The thicknesses of individual layers were measured with a surface profilometer. The 2D-GIXD data were acquired at station 14B in Shanghai Synchrotron Radiation Facility.

## Results and discussion

**Figure 1:**
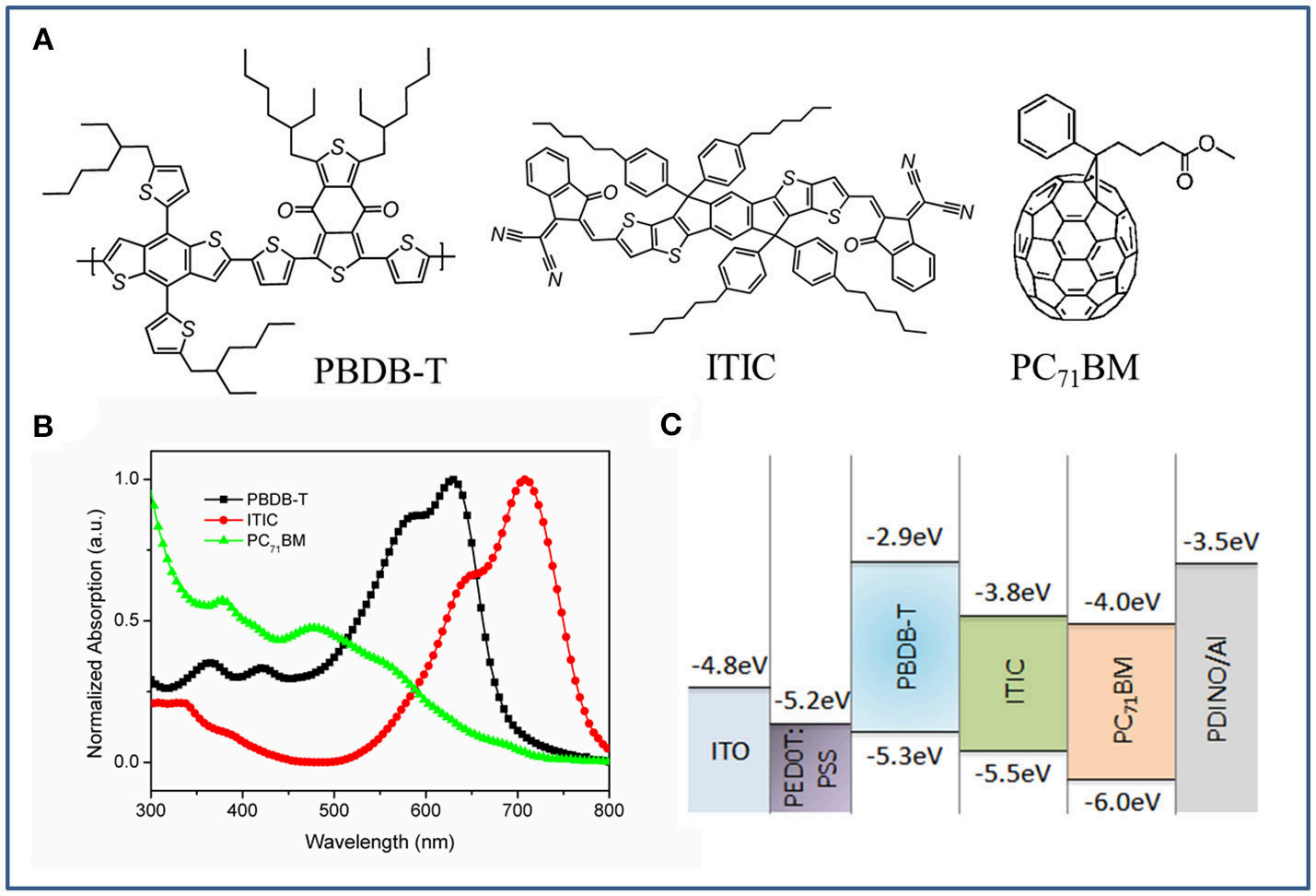
**(A)** Chemical structures of PBDB-T, ITIC, and PC_71_BM. **(B)** Normalized absorption of pure PBDB-T, ITIC, and PC_71_BM films. **(C)** Energy level diagram of solar cells.

The chemical structures of PBDB-T, ITIC and PC_71_BM are shown in Figure 1A. The normalized UV-vis absorption spectra of neat PBDB-T, ITIC and PC_71_BM films are plotted in Figure 1B. It is clearly seen from Figure 1B that the main peaks of PBDB-T and ITIC are located at 625 and 710 nm, respectively, and their complementary absorption can strongly improve the spectral coverage in the visible region. PC_71_BM exhibits absorption in short wavelength region but its absorption capability is relatively weak. In this study, the PSCs with a conventional device structure of ITO/PEDOT:PSS/active layer/PDINO/Al were fabricated, and their energy levels were plotted in Figure 1C. The polymer PDINO was used as cathode buffer layer referenced to the literature (Zhang Z. et al., 2014). The total ratio of donor component to acceptor component is kept at a fixed ratio of 1:1 (w/w). The ratio of PBDB-T:ITIC:PC_71_BM is marked as the D:A1:A2.

**Figure 2:**
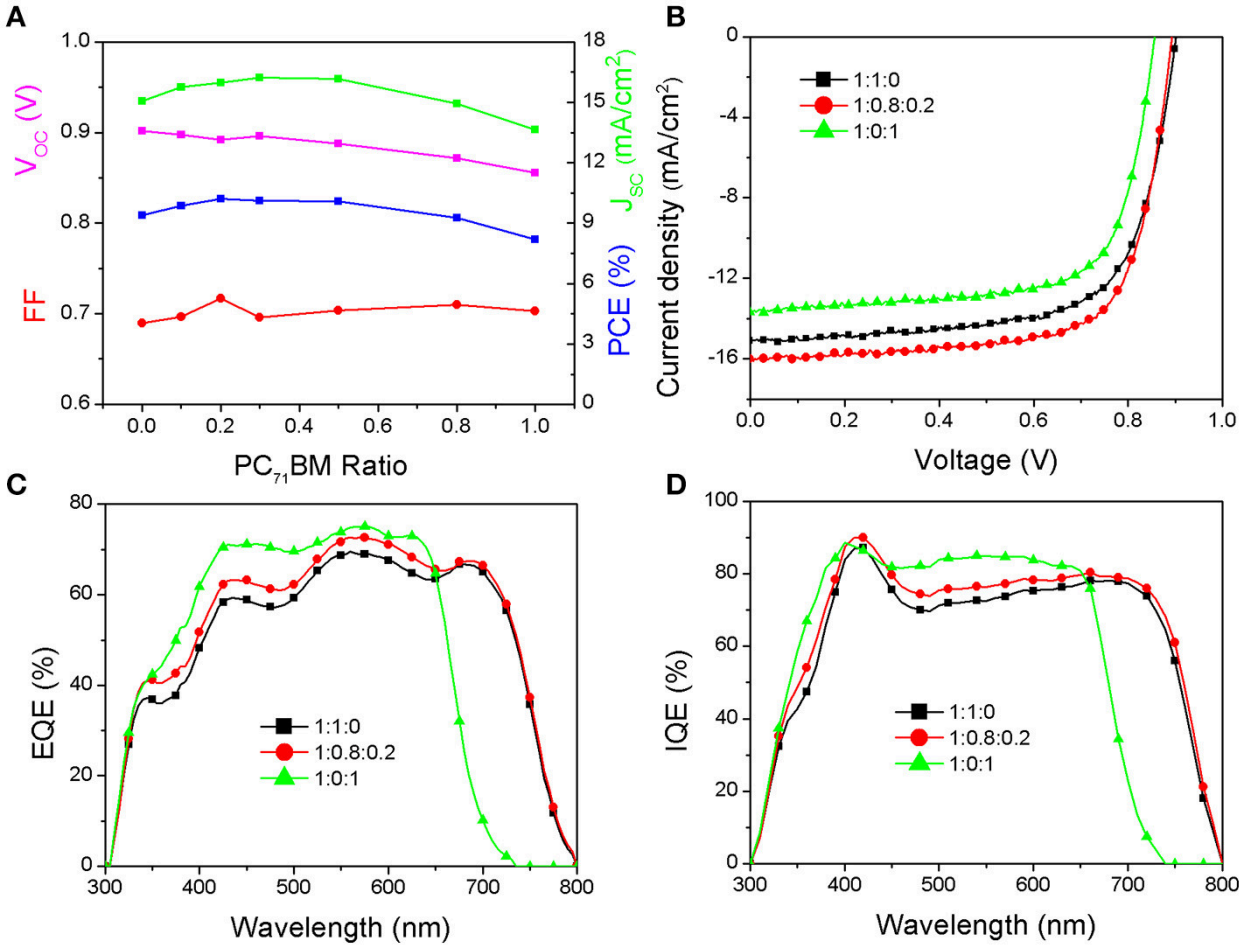
**(A)** The photovoltaic parameters of the PBDB-T:ITIC:PC_71_BM (1:1-x:x) PSCs. The J-V **(B)**, EQE **(C)**, and IQE **(D)** curves of PSCs based on PBDB-T:ITIC (1:1), PBDB-T:ITIC:PC_71_BM (1:0.8:0.2), and PBDB-T:PC_71_BM (1:1) blend films, respectively.

**Figure 3:**
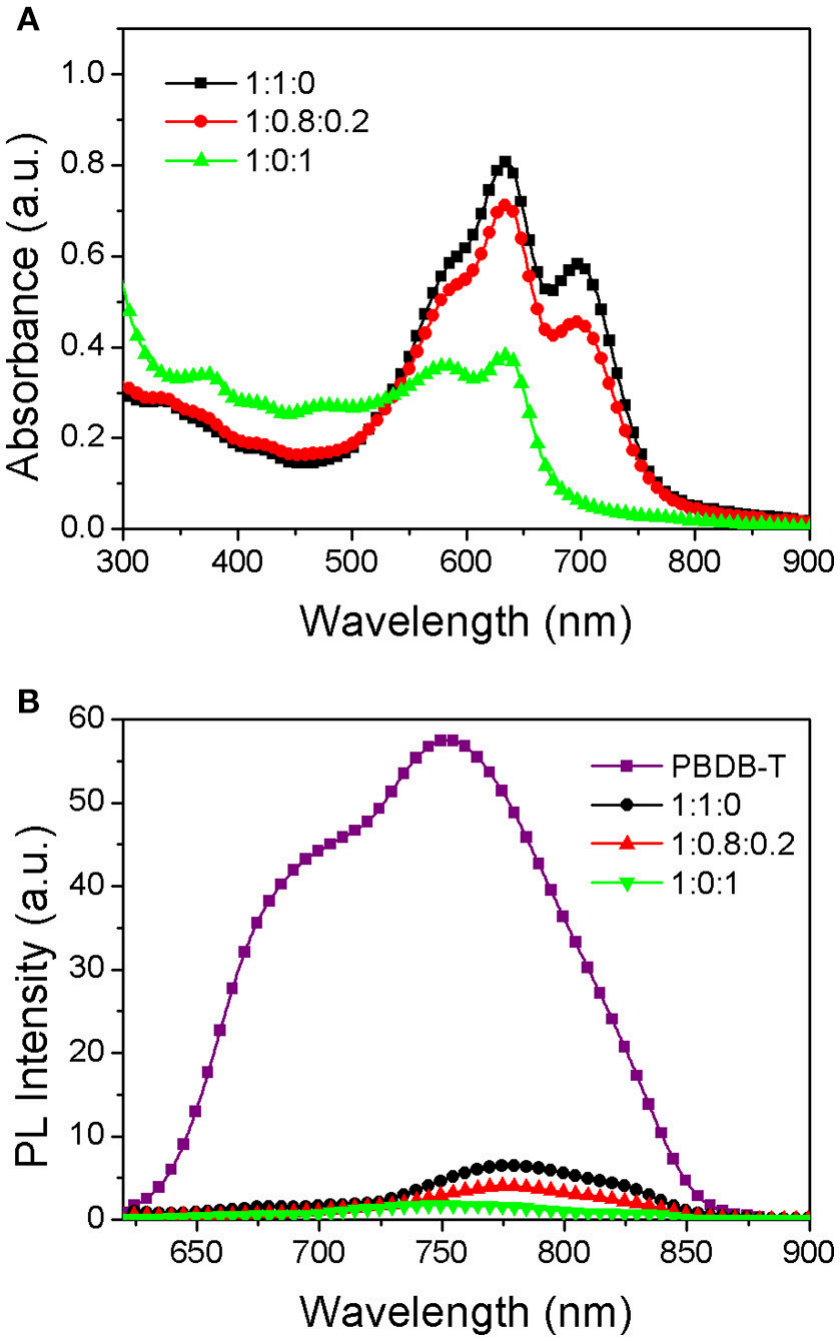
**(A)** Absolute absorption spectra of PBDB-T:ITIC (1:1), PBDB-T:ITIC:PC_71_BM (1:0.8:0.2), and PBDB-T:PC_71_BM (1:1) blend films. **(B)** Photoluminescent spectra of pure PBDB-T, PBDB-T:ITIC (1:1), PBDB-T:ITIC:PC_71_BM (1:0.8:0.2), and PBDB-T:PC_71_BM (1:1) films.

The photovoltaic performance of the PSCs based on the PBDB-T:ITIC:PC_71_BM (1:1-x:x) blend were firstly evaluated. The ratio of A1:A2 is varied from 1:0 to 0:1 while the ratio of D:(A1+A2) is fixed at 1:1 in order to keep a constant active layer thickness. The detailed photovoltaic parameters of the resultant PSCs with different ratio of two acceptors are listed in Table S1 in Supplementary Information. The dependence of V_OC_, J_SC_, FF, and PCE on the PC_71_BM content for the resultant PSCs are plotted in Figure 2A. In the case of V_OC_, it undergoes gradually decrease from 0.902 V for the binary PBDB-T:ITIC PSCs to 0.856 V for the binary PBDB-T: PC_71_BM PSCs with increasing PC_71_BM content from 0 to 1 in the ternary blend PSCs. The change is reasonable since the LUMO of ITIC is higher than that of PC_71_BM, and the electron transport and collection will occur in PC_71_BM phases when the PC_71_BM amount is larger. The interesting thing is that the J_SC_ are initially increased and then decreased with the increase of PC_71_BM contents for the resultant ternary PSCs. The FF shows similar trend. Considering the intrinsic absorption properties between ITIC and PC_71_BM, the substitution of small amount of ITIC by PC_71_BM would decrease the total absorption of the ternary PBDB-T:ITIC:PC_71_BM blend active layer. Thus, the improved J_SC_ and FF for the ternary PBDB-T:ITIC:PC_71_BM PSCs may imply that both the exciton dissociation and charge-collection efficiencies are enhanced in comparison to the binary PBDB-T:ITIC PSCs. It is plotted the illuminated J-V curves of the PBDB-T:ITIC PSCs, the PBDB-T:PC_71_BM PSCs and the optimized ternary PBDB-T:ITIC:PC_71_BM PSCs in Figure 2B, respectively. The non-fullerene PSCs based on binary PBDB-T:ITIC blend demonstrate a V_OC_ of 0.902 V, a J_SC_ of 15.06 mA/cm^2^, a FF of 0.69, respectively, leading to a PCE of 9.38%. Due to the limited spectral coverage, the PBDB-T:PC_71_BM PSCs show a lower J_SC_ of 13.64 mA/cm^2^, and finally result in a PCE of 8.21% together with a V_OC_ of 0.856 V and a FF of 0.703. The ternary PBDB-T:ITIC:PC_71_BM (1:0.8:0.2) PSCs demonstrate a V_OC_ of 0.892 V, a J_SC_ of 15.98 mA/cm^2^, a FF of 0.717 and an overall PCE of 10.22%. Although the V_OC_ is a little lowered in comparison to the binary PBDB-T:ITIC PSCs, the increased J_SC_ and FF boost the PCE enhancement of the ternary PSCs. The external quantum efficiency (EQE) curves of the three devices are shown in Figure 2C. The spectral response of the PBDB-T:PC_71_BM PSCs cover at a range of 300–700 nm with EQE higher than 70% at 450–650 nm. The PBDB-T:ITIC PSCs show extended spectral coverage of 300–800 nm due to the narrow bandgap of ITIC. The ternary PBDB-T:ITIC:PC_71_BM PSCs show similar spectral response profile but high EQE compared to the PBDB-T:ITIC PSCs. The internal quantum efficiency (IQE) of these devices are also measured to clarify the absolute quantum efficiency in these devices and the curves are plotted in Figure 2D. It indicates that the photon-to-electron conversion efficiency is really improved by adding some amount of PC_71_BM into the PBDB-T:ITIC blend. The absorption spectra of the active layers in three kinds of PSCs are plotted in Figure 3A. The PBDB-T:ITIC:PC_71_BM (1:0.8:0.2) active layer shows a little low absorption at 550–750 nm due to decreased ITIC content compared to the PBDB-T:ITIC (1:1) film. Although the PBDB-T:PC_71_BM (1:1) film demonstrates enhanced absorption at 300–500 nm, its absorption at 550–700 nm is remarkably decreased. Photoluminescence (PL) quenching experiments were carried out to check the charge transfer status in these films. As shown in Figure 3B, the introduction of PC_71_BM favors to quench the excitons dominated on the PBDB-T donor.

**Figure 4:**
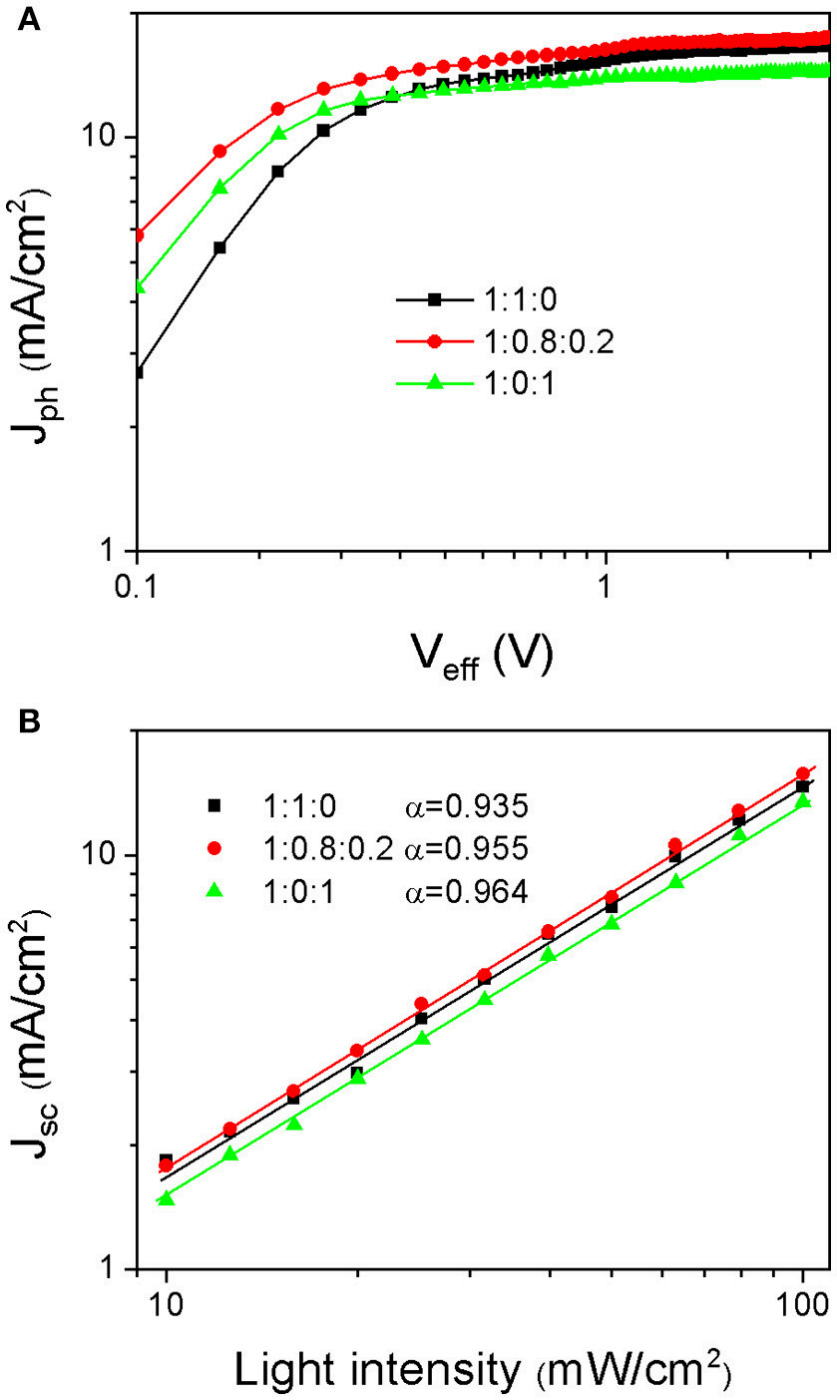
**(A)** J_ph_ – V_eff_ curves and **(B)** J_SC_ dependence on the illuminated light intensity of PSCs based on PBDB-T:ITIC (1:1), PBDB-T:ITIC:PC_71_BM (1:0.8:0.2), and PBDB-T:PC_71_BM (1:1) blend films, respectively.

The relationship between photocurrent (J_ph_) and effective voltage (V_eff_) is investigated to judge the charge generation and collection status in the PSCs based on the PBDB-T:ITIC (1:1), PBDB-T:ITIC:PC_71_BM (1:0.8:0.2), and PBDB-T:PC_71_BM (1:1) blends (Yuan et al., 2017; Zhang G. et al., 2017). As shown in Figure 4A, J_ph_ is given by J_ph_ = J_L_ − J_D_, where J_L_ & J_D_ are the current density under illumination and in the dark, respectively. V_eff_ is defined as V_0_ − V_appl_, V_0_ is the voltage when J_ph_ = 0 and V_appl_ is the applied voltage during the measurement. The J_ph_ is supposed to be saturated at a V_eff_ of 3 V, which are 16.55 mA/cm^2^, 17.40 mA/cm^2^ and 14.45 mA/cm^2^, respectively, for the PSCs based on the PBDB-T:ITIC (1:1), PBDB-T:ITIC:PC_71_BM (1:0.8:0.2), and PBDB-T:PC_71_BM (1:1) blends. The device based on ternary PBDB-T:ITIC:PC_71_BM (1:0.8:0.2) blend demonstrates the highest saturation current, indicating its highest charge-generation capability. Charge dissociation probability P(E,T) is defined as J_ph_/J_sat_. When a high bias is applied, the charge recombination is suppressed and most of photo-generated charges are extracted, leading to P(E,T) close to100%. It is calculated that the P(E,T) values under short-circuit condition (V_appl_ = 0 V) are 90.6, 91.9, and 94.9%, respectively, for the PSCs based on the PBDB-T:ITIC (1:1), PBDB-T:ITIC:PC_71_BM (1:0.8:0.2), and PBDB-T:PC_71_BM (1:1) blends. The PBDB-T:PC_71_BM (1:1) device shows the best charge-extraction capability, whereas the charge-extraction ability is a little poor for the PBDB-T:ITIC (1:1) device. The ternary PBDB-T:ITIC:PC_71_BM (1:0.8:0.2) shows improved exciton dissociation and charge extraction compared to the PBDB-T:ITIC (1:1) device. The charge recombination status in these devices is also investigated via the dependence of J_SC_ on the incident light intensity as shown in Figure 4B. J_SC_ is dependent on the incident light intensity with J_SC_∝I^α^, in which exponential factor α would be close to unity without bimolecular recombination (Gao et al., 2016; Xu et al., 2017, 2018). The PBDB-T:PC_71_BM (1:1) device has the highest α value of 0.964, whereas α value of the PBDB-T:ITIC (1:1) device is merely 0.935 implying severe bimolecular recombination within the film. Such kind of bimolecular recombination in the PBDB-T:ITIC (1:1) device is to some extent suppressed with α value of 0.955 in ternary PBDB-T:ITIC:PC_71_BM (1:0.8:0.2) device.

As discussed above, with the introduction of PC_71_BM into the PBDB-T:ITIC blend, the resultant ternary PSCs demonstrate an enhanced exciton-dissociating and charge-extracting property, leading to improved PCE compared to the PBDB-T:ITIC PSCs. The detailed mechanism accounting for the enhancement is further investigated. The electron and hole transport properties of the various blend films were firstly measured via space-charge limited current (SCLC) method (Bin et al., 2016). The J-V curves of the devices were plotted as in Figure S1 and the calculated electron and hole mobility are listed in Table S2. The PBDB-T:PC_71_BM (1:1) blend film shows relatively high electron and hole mobility of 8.45 × 10^−4^ and 5.30 × 10^−4^ cm^2^V^−1^s^−1^, respectively. However, the electron and hole mobility of the PBDB-T:ITIC (1:1) film is 3.05 × 10^−4^ and 2.70 × 10^−4^ cm^2^V^−1^s^−1^. After incorporating PC_71_BM, the electron and hole mobility of the resultant PBDB-T:ITIC:PC_71_BM (1:0.8:0.2) film is increased to 4.55 × 10^−4^ and 3.37 × 10^−4^ cm^2^V^−1^s^−1^. As it is known, the high and balanced charge transport may help to efficient charge extraction and hence low charge recombination. This is in agreement with the recombination status in these PSCs. It is speculated that the incorporation of PC_71_BM improves the PBDB-T and ITIC interpenetrating networks and thus the photovoltaic performance.

**Figure 5:**
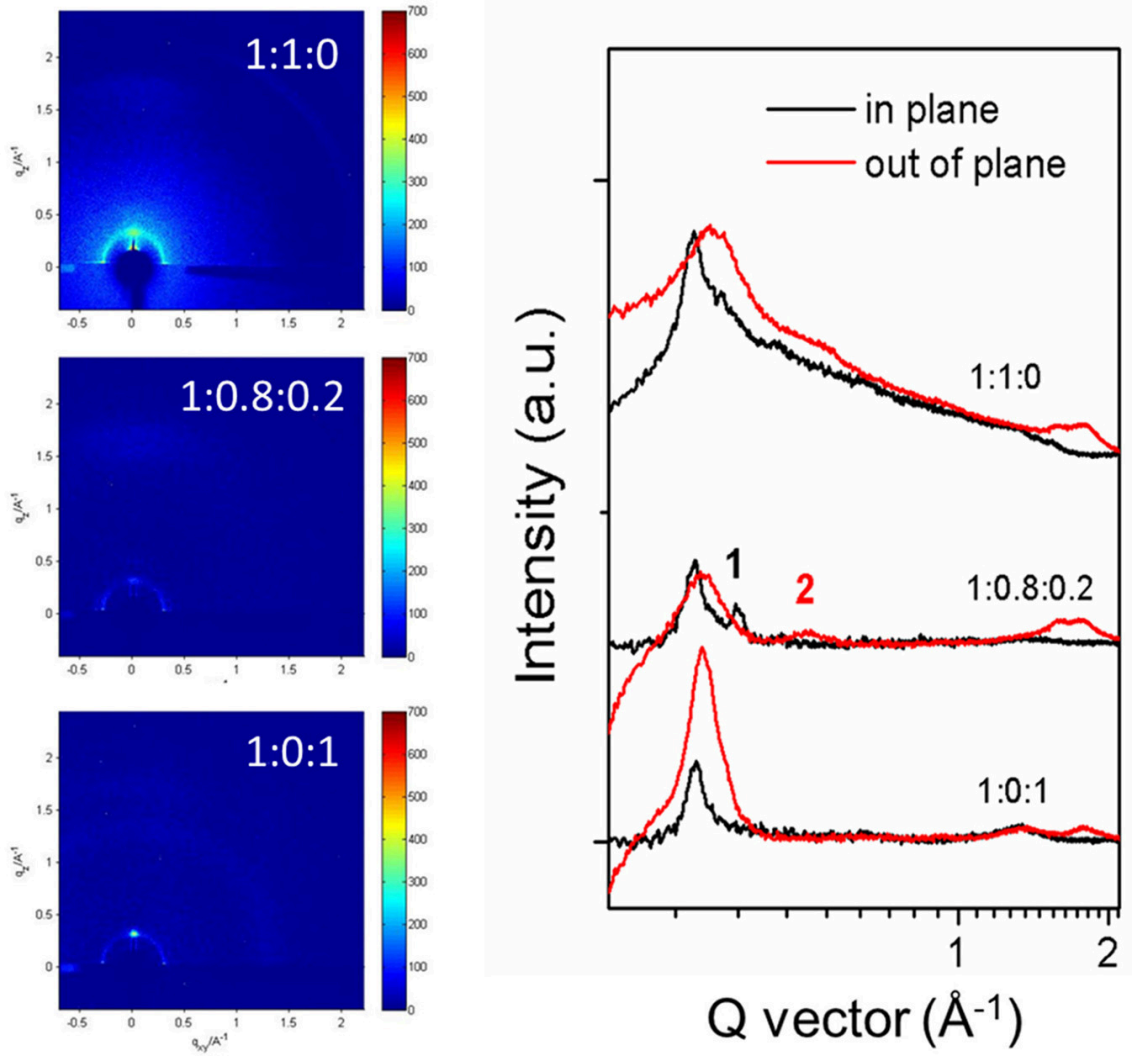
2D-GIXD images of PBDB-T:ITIC (1:1), PBDB-T:ITIC:PC_71_BM (1:0.8:0.2), and PBDB-T:PC_71_BM (1:1) blend films **(Left)**. The in-plane and out-of-plane line profiles of the films **(Right)**.

The morphology of the PBDB-T:ITIC (1:1), PBDB-T:ITIC:PC_71_BM (1:0.8:0.2), and PBDB-T:PC_71_BM (1:1) blend films are investigated and their AFM and TEM images are shown in Figures S2, S3. All the blend films are smooth with a root mean square (RMS) roughness of <2 nm. However, the morphology of the ternary blend is not changed obviously compared to the PBDB-T:ITIC (1:1) blend film. The structural information of the blended films are further investigated by grazing incidence X-ray diffraction (GIXD). The 2D-GIXD images of the pure PBDB-T and ITIC films are plotted in Figure S4. The pure PBDB-T film shows dominant peak at q_xy_ = 0.29 Å^−1^ in the in-plane direction, attributing to its strong (100) diffraction. The pure ITIC film demonstrates two featured peaks at q_z_ = 0.26 Å^−1^ and q_z_ = 0.53 Å^−1^ in out-of-plane direction and one peak in in-plane direction at q_xy_ = 0.36 Å^−1^. 2D-GIXD patterns and line-cut profiles of the PBDB-T:ITIC (1:1), PBDB-T:ITIC:PC_71_BM (1:0.8:0.2) and PBDB-T:PC_71_BM (1:1) films are shown in Figure 5. The diffractions originated from PBDB-T aggregation is observed both in PBDB-T:ITIC (1:1) and PBDB-T:PC_71_BM (1:1) films in contrast to the pure PBDB-T film. However, the diffraction signal originated from the ITIC component is not obviously presented in PBDB-T:ITIC blend film, implying its poor aggregation. In the PBDB-T:ITIC:PC_71_BM (1:0.8:0.2) blend film, the diffraction signal from PBDB-T is still presented but weakened. More importantly, two additional peaks marked 1 and 2 are presented in the ternary blend film compared to the PBDB-T:ITIC (1:1) counterpart, which corresponds to the featured diffraction from ITIC in the in-plane and out-of-plane directions. Bo et al. observed similar phenomenon in their work (Lu et al., 2016). This indicates that the ITIC aggregation is enhanced in the PBDB-T:ITIC:PC_71_BM (1:0.8:0.2) blend film though the ITIC content is decreased. In other words, the small amount of PC_71_BM may serve as “lubricant” to favor ITIC molecules to aggregate out of the polymer PBDB-T matrix.

**Figure 6:**
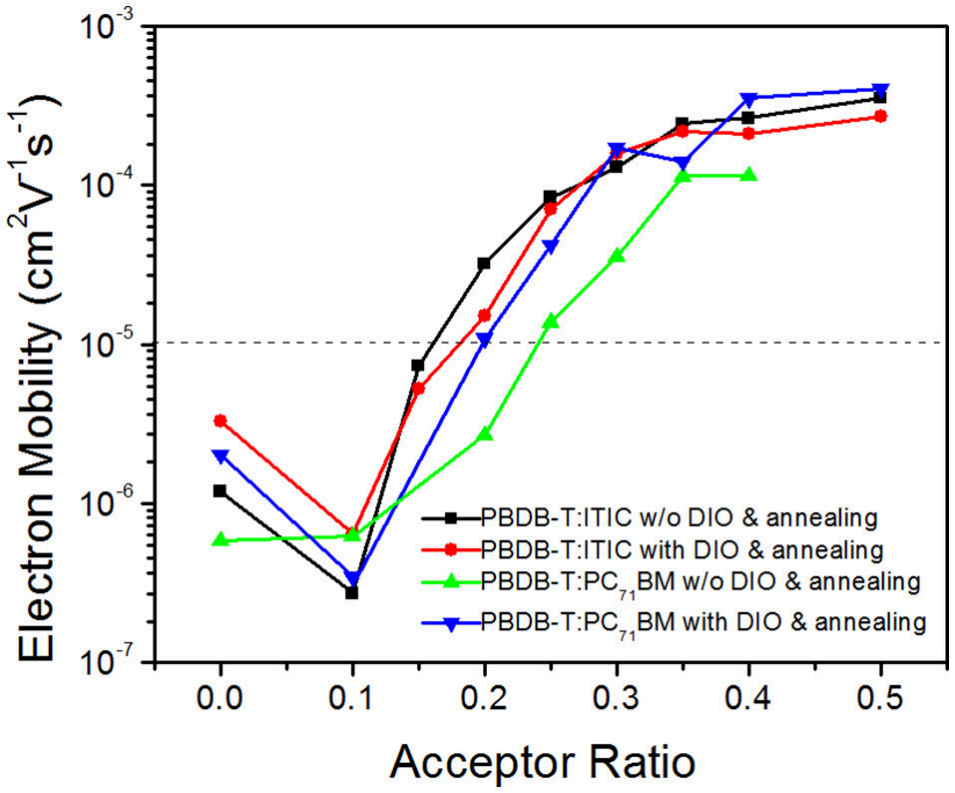
The measured electron mobility in the PBDB-T:ITIC (1:x) and PBDB-T:PC_71_BM (1:x) films.

The prerequisite to get such a conclusion is that the compatibility between PBDB-T and PC_71_BM should be better than that between PBDB-T and ITIC. The electron mobility of the PBDB-T:ITIC (1:x) and PBDB-T:PC_71_BM (1:x) blend films are measured and its dependence on the acceptor ratio is plotted in Figure 6. It is supposed that the acceptor phase forms continuous tunnels when the electron mobility of the blend films exceeds 10^−5^ cm^2^V^−1^s^−1^. Both the as-prepared film deposited in CB solution and the annealed film deposited in CB:DIO (0.5%, v/v) solution are tested. The results show that the continuous electron-transport tunnels is formed in the PBDB-T:ITIC film with the ITIC/PBDB-T ratio <20%. The DIO additive and thermal annealing donot change the electron transport remarkably. For the as-prepared PBDB-T:PC_71_BM film, the critical PC_71_BM content is increased to 25%. The DIO additive and thermal annealing lowers the critical PC_71_BM content. This indicates that the PBDB-T is inclined to intermix with PC_71_BM better than with ITIC. In the ternary PBDB-T:ITIC:PC_71_BM (1:0.8:0.2) blend film, the small amount of PC_71_BM may mix with PBDB-T and does not form networks. Moreover, the existence of PC_71_BM favors ITIC aggregating to form electron-transporting networks. This is confirmed by the increased electron mobility and the XRD results compared to the PBDB-T:ITIC (1:1) film.

**Figure 7:**
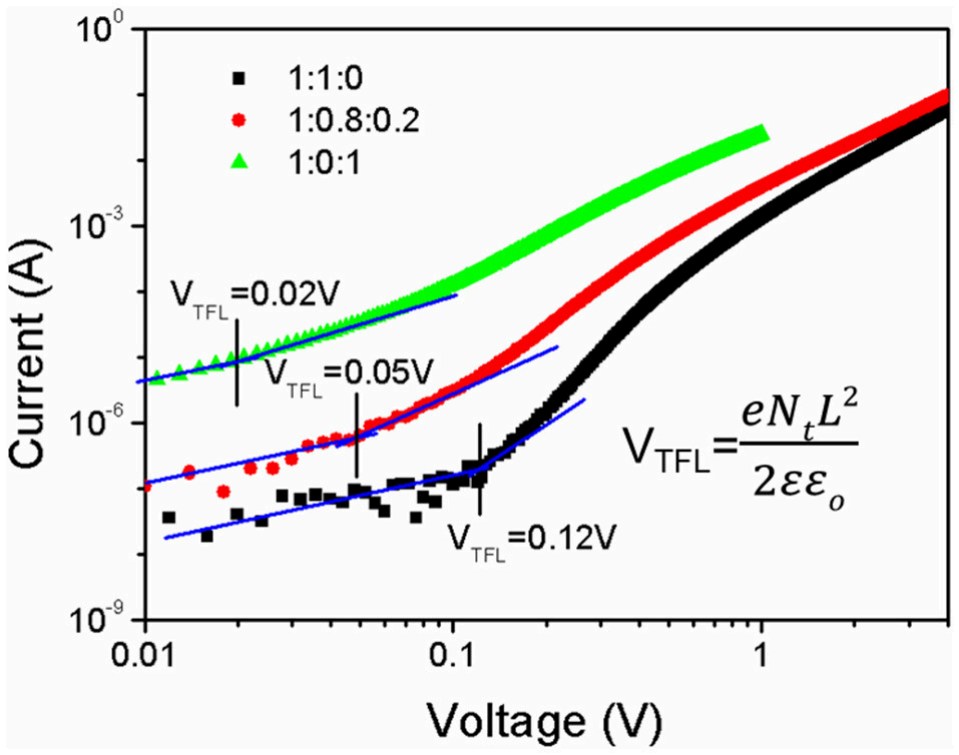
The current-voltage curves of the electron-only devices based on the PBDB-T:ITIC (1:1), PBDB-T:ITIC:PC_71_BM (1:0.8:0.2), and PBDB-T:PC_71_BM (1:1) blend films.

The electron trap-state density in these blend films is further investigated. From the I–V curves of the electron-only devices based on the PBDB-T:ITIC (1:1), PBDB-T:ITIC:PC_71_BM (1:0.8:0.2) and PBDB-T:PC_71_BM (1:1) blend shown in Figure 7, the electron trap density N is calculated by the equation as below (Yang et al., 2016):

(1) $$\begin{array}{l} V_{TFL}=\frac{eNL^{2}}{2\varepsilon_{0}\varepsilon_{r}} \end{array}$$

where V_TFL_ is the trap-filled limit voltage, e is the elementary charge of electron, L is the thickness of film, ϵ_0_ is the vacuum permittivity, and ϵ_r_ is the relative dielectric constant (ϵ_r_ = 3). The V_TFL_ of three devices is 0.12, 0.05, and 0.02 V. The calculated deep trap density is 3.3 × 10^15^, 1.5 × 10^15^, and 7.3 × 10^14^ cm^−3^, respectively. It confirms that the incorporation of PC_71_BM is helpful for ITIC molecules to move out of PBDB-T phases and thus the density of electron traps is reduced. The increased electron mobility and the decreased electron traps are helpful to enhance the photovoltaic performance of the PBDB-T:ITIC:PC_71_BM (1:0.8:0.2).

## Conclusion

In summary, the ternary PSCs based on PBDB-T:ITIC:PC_71_BM (1:0.8:0.2) blend were fabricated and its PCE was increased to 10.2% compared to 9.2% for the PBDB-T:ITIC (1:1) devices. The mechanism accounting for the enhanced photovoltaic performance is discussed in detail. It is found that the PC_71_BM tends to intermix with the PBDB-T donor compared to the ITIC counterpart. A small amount of PC_71_BM in the ternary blend is helpful for ITIC to aggregate and form efficient electron-transport pathways. The electron mobility is increased and the density of electron traps is decreased in the ternary PBDB-T:ITIC:PC_71_BM (1:0.8:0.2) blend in comparison with the PBDB-T:ITIC blend. Finally, the suppressed bimolecular recombination and enhanced charge collection lead to a high PCE for the ternary solar cells.

## Author contributions

BW and CY conceived and designed the experiments. BW performed the experiments. RZ performed 2D-GIXD measurements. QY performed the absorption and PL experiments. BW and YF analyzed data. BW wrote the manuscript. All authors discussed and commented on the paper.

### Conflict of interest statement

The authors declare that the research was conducted in the absence of any commercial or financial relationships that could be construed as a potential conflict of interest.
