# Supplementary material for: Insight Into the Role of PC71BM on Enhancing the Photovoltaic Performance of Ternary Organic Solar Cells
Source: Front Chem. 2018 Jun 5;6:198. doi: 10.3389/fchem.2018.00198 (PMC5996040; doi:10.3389/fchem.2018.00198)
Supplement: Supplementary file 1 [file Image_1.PDF]

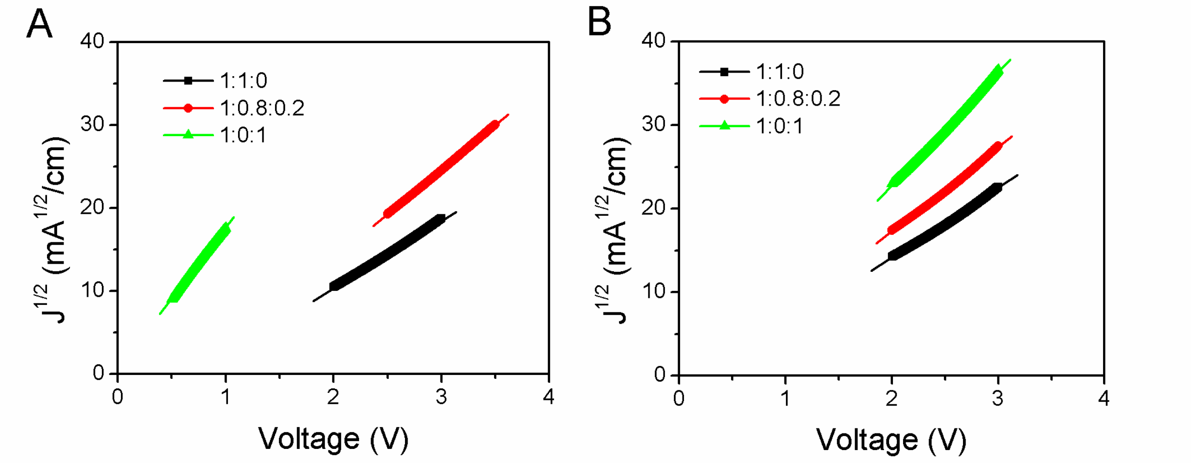

Figure S1. $J^{1/2}$-V_{appl} curves of the electron-only (A) and hole-only devices (B) fitted with the SCLC model.
