# Supplementary material for: Insight Into the Role of PC71BM on Enhancing the Photovoltaic Performance of Ternary Organic Solar Cells
Source: Front Chem. 2018 Jun 5;6:198. doi: 10.3389/fchem.2018.00198 (PMC5996040; doi:10.3389/fchem.2018.00198)
Supplement: Supplementary file 3 [file Image_3.PDF]

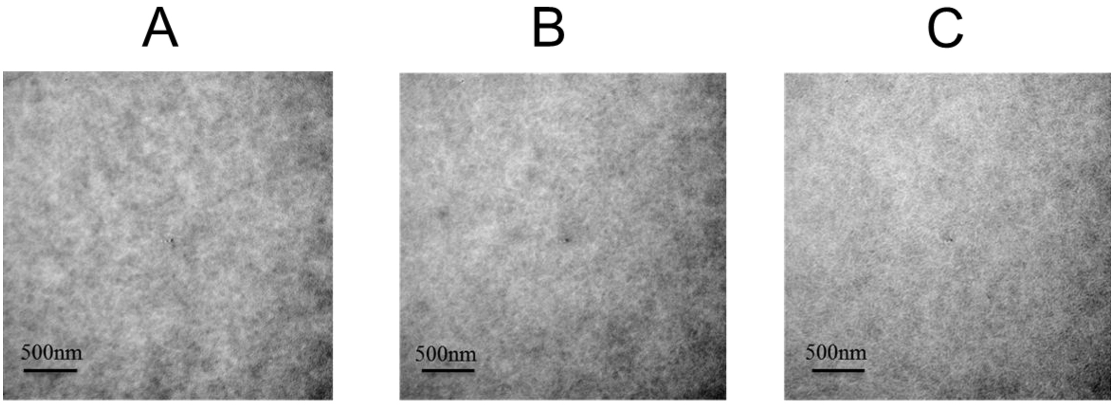

Figure S3. TEM images of the (A) PBDB-T:ITIC (1:1), (B) PBDB-T:ITIC:$PC_{71}BM$ (1:0.8:0.2) and (C) PBDB-T:$PC_{71}BM$ (1:1) blend films.
