# Supplementary figures and images for: Insight Into the Role of PC71BM on Enhancing the Photovoltaic Performance of Ternary Organic Solar Cells
Source: Front Chem. 2018 Jun 5;6:198. doi: 10.3389/fchem.2018.00198 (PMC5996040; doi:10.3389/fchem.2018.00198)

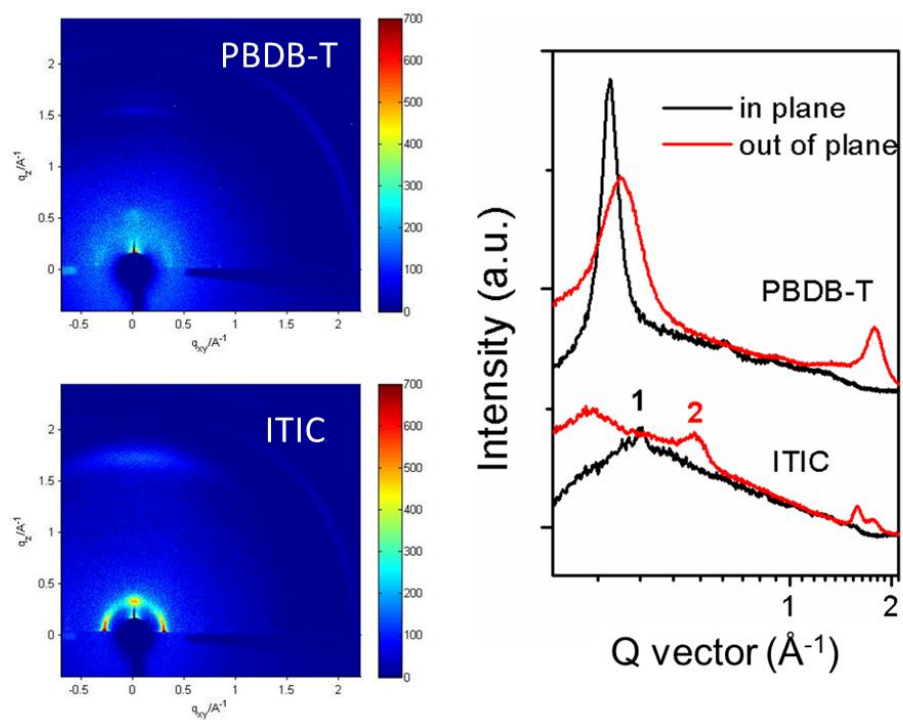

**Figure S4.** The 2D-GIXD images of pure PBDB-T and ITIC films (left) and line profiles of them (right).

Supplement: Supplementary file 4 [file Image_4.PDF]
