# Supplementary material for: Insight Into the Role of PC71BM on Enhancing the Photovoltaic Performance of Ternary Organic Solar Cells
Source: Front Chem. 2018 Jun 5;6:198. doi: 10.3389/fchem.2018.00198 (PMC5996040; doi:10.3389/fchem.2018.00198)
Supplement: Supplementary file 5 [file Table_1.PDF]

Table S1. The detailed photovoltaic parameters of binary control devices and ternary devices with different acceptor weight ratios. (Averaged values are calculated from more than 8 devices.)

PBDBT:ITIC :$PC_{71}BM$	V_{OC} (V)	J_{SC} (mA/cm^2)	FF	PCE[avg.] (%)	J_{SC}[cal.] (mA/cm^2)
1:1:0	0.902	15.06	0.690	9.38[9.18]	15.02
1:0.95:0.05	0.898	15.64	0.708	9.94[9.89]	15.14
1:0.9:0.1	0.898	15.76	0.697	9.86[9.78]	15.33
1:0.85:0.15	0.894	15.91	0.699	9.94[9.85]	15.52
1:0.8:0.2	0.892	15.98	0.717	10.22[10.21]	15.72
1:0.7:0.3	0.896	16.24	0.696	10.12[9.96]	15.68
1:0.5:0.5	0.888	16.16	0.704	10.10[10.01]	15.80
1:0.2:0.8	0.872	14.93	0.710	9.25[9.12]	14.46
1:0:1	0.856	13.64	0.703	8.21[8.08]	13.05
