# Supplementary material for: Insight Into the Role of PC71BM on Enhancing the Photovoltaic Performance of Ternary Organic Solar Cells
Source: Front Chem. 2018 Jun 5;6:198. doi: 10.3389/fchem.2018.00198 (PMC5996040; doi:10.3389/fchem.2018.00198)
Supplement: Supplementary file 6 [file Table_2.PDF]

Table S2. The hole and electron mobilities of the blend films calculated from their electron-only and hole-only devices.

PBDB-T:ITIC:$PC_{71}BM$	Electron mobility ($10^{-4}cm^2V^{-1}s^{-1}$)	Hole mobility ($10^{-4}cm^2V^{-1}s^{-1}$)
1:1:0	3.05	2.70
1:0.8:0.2	4.55	3.37
1:0:1	8.45	5.30
